# Chemoprevention of dietary digitoflavone on colitis-associated colon tumorigenesis through inducing Nrf2 signaling pathway and inhibition of inflammation

**DOI:** 10.1186/1476-4598-13-48

**Published:** 2014-03-06

**Authors:** Yang Yang, Xueting Cai, Jie Yang, Xiaoyan Sun, Chunping Hu, Zhanpeng Yan, Xiaojun Xu, Wuguang Lu, Xiaoning Wang, Peng Cao

**Affiliations:** 1 Laboratory of Cellular and Molecular Biology, Jiangsu Province Institute of Traditional Chinese Medicine, 100#, Shizi Street, Hongshan Road, Nanjing 210028, Jiangsu, China; 2 State Key Laboratory of Natural Medicines, China Pharmaceutical University, 24 Tongjiaxiang, Nanjing 210009, Jiangsu, China

**Keywords:** Digitoflavone, Luteolin, Reactive oxygen species (ROS), Nrf2, p38 MAPK, Chemoprevention

## Abstract

**Background:**

Nuclear factor-erythroid 2-related factor 2 (Nrf2) has emerged as a novel target for the prevention of colorectal cancer (CRC). Many chemopreventive compounds associated with Nrf2 activation are effective in preclinical systems and many on-going clinical trials are showing promising findings. In present study we evaluated the cytoprotective effect and chemopreventive properties of dietary digitoflavone.

**Method:**

A cell based Antioxidant Response Element (ARE)-driven luciferase reporter system was applied to screen potential Nrf2 activators. Activation of Nrf2 by digitoflavone was confirmed through mRNA, protein and GSH level assay in Caco-2 cell line. The cytoprotective effect of digitoflavone was evaluated in H_2_O_2_-induced oxidative stress model and further signaling pathways analysis was used to determine the target of digitoflavone induced Nrf2 activation. An AOM-DSS induced colorectal cancer model was used to assess the chemopreventive effect of digitoflavone.

**Result:**

Micromolarity (10 μM) level of digitoflavone increased Nrf2 expressing, nuclear translocation and expression of downstream phase II antioxidant enzymes. Furthermore, digitoflavone decreased H_2_O_2_-induced oxidative stress and cell death via p38 MAPK-Nrf2/ARE pathway. *In vivo* study, 50 mg/kg digitoflavone significantly reduced AOM-DSS induced tumor incidence, number and size.

**Conclusion:**

These observations suggest that digitoflavone is a novel Nrf2 pathway activator, and protects against oxidative stress-induced cell injury. The results of the present study add further evidence of the molecular mechanisms that allow digitoflavone to exert protective effects and reaffirm its potential role as a chemopreventive agent in colorectal carcinogenesis.

## Introduction

Gastrointestinal tract is body’s digestion and absorption organ and frequently faces the challenges from xenobiotics and endogenous toxic substances-induced oxidative stress due to its particular location and function. Also, Reactive Oxygen Species (ROS) are involved in many physiological functions and colorectal pathological processes, such as Crohn’s disease, ulcerative colitis, and colorectal cancer (CRC) [1-3]. Therefore, there is an increasing interest in the potential effects of exogenous antioxidants on the prevention of oxidative gastrointestinal disorders. Recently, Up-regulation of endogenous antioxidant and phase II antioxidant enzymes by Nrf2 has emerged as a novel target for the prevention of CRC since it is currently well accepted that chronic inflammation is a contributing factor in 15-20% malignancies including CRC [4] and that this inflammation can be attributed to a number of factors including oxidative stress, reactive oxygen species (ROS) and reactive nitrogen species (RNS).

Phase II metabolizing-detoxifying and antioxidant defense enzymes [such as NADP(H) quinone oxidoreductase 1 (NQO-1), heme oxygenase-1 (HO-1), and aldo-keto reductase1 subunits C-1 ( AKR1C1), C-2 ( AKR1C2) and C-3 (AKR1C3) ] , antioxidants [such as γ-glutamylcysteine synthetase catalytic subunit(γ-GCSc) , γ-glutamylcysteine synthetase modifier subunit (γ-GCSm), glutathione reductase (GR), thioredoxin reductase (TR) and peroxiredoxin (Prx)], and ATP-dependent drug efflux pumps [ATP- binding cassette, subfamily C(CFTR/MRP) members (ABCC2, ABCC3, ABCC4)] are regulated by cis-acting regulatory element-the antioxidant responsive element (ARE; 5′-(A/G) TGACNNNGC(A/G) -3′) [5], and Nrf2, a member of the Cap’n’ Collar family of transcription factors, which mainly regulates transcriptional activation through the ARE [6]. The Nrf2/ARE signal pathway has been considered to protect cells against carcinogenesis and attenuate cancer development via neutralization of ROS or carcinogens [7,8]. Nrf2-deficient mice were more susceptible to carcinogenesis, suggesting that Nrf2/ARE mediates the phase II detoxifying enzymes and antioxidant proteins in the inactivation of chemical carcinogens [9].

**Figure 1:**
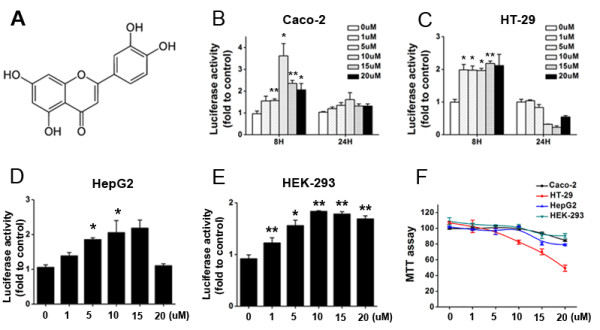
**Effects of digitoflavone on ARE–luciferase activity. (A) **Chemical structure of digitoflavone (3, 0, 4, 5, 7-tetrahydroxyflavone). Its molecular weight is 286.23. **(B-C)** Effects of digitoflavone on ARE–luciferase activity in Caco-2 and HT-29 cells. Caco-2 cells were treated with various concentrations of digitoflavone for 8 hours and 24 hours respectively, luciferase activity was assayed as described in Materials and Methods. **(D-E)** HEK-293 cells, HepG2 cells were treated with various concentrations of digitoflavone for 8 hours and luciferase activity were assayed as described in Materials and Methods. **(F)** Cell viability of digitoflavone in Caco-2, HT-29, HepG2 and HEK-293 cells. Cells were treated with various concentrations (1–20 μM) of digitoflavone for 24 hours, cell viability were determined using MTT assay. Values were expressed as mean ± SD of triplicate experiments. Significantly different (versus control group): *p<0.05 and **p<0.01.

Functional foods act as antioxidant nutrients and protect against many human chronic diseases by combating reactive oxygen species (ROS) generation [10,11]. As diet antioxidants, flavonoids, polyphenolic compounds occurring naturally in the plant kingdom such as vegetables, fruits and plant-derived beverages such as tea, cocoa, and red wine, display a wide range of pharmacological properties, including anti-carcinogenesis and anti-inflammation [12]. Flavonoids also exert a potent antioxidant activity, acting as reactive oxygen species (ROS) scavengers, metal ions chelators and free radical reaction terminators [13]. However, they can also act indirectly as antioxidants stimulating phase II detoxifying and antioxidant defense enzymes to preserve cellular integrity and tissue homeostasis [14]. Digitoflavone (3, 0, 4, 5, 7-tetrahydroxyflavone, Figure 1A), a flavone subclass of flavonoids, vegetables and fruits such as celery, parsley, broccoli, onion leaves, carrots, peppers, cabbages, apple skins, and chrysanthemum flowers are digitoflavone rich [15-18]. Plants rich in digitoflavone have been used as Chinese traditional medicine for hypertension, inflammatory diseases, and cancer [19]. Also, it has been known to have chemopreventive effects against malignant tumors *in vivo*[20,21]. Our recent study has found digitoflavone induce G2 phase cell cycle arrest, inhibit angiogenesis and down-regulate expression of NF-κB [22-24]. Much attention has been focused on digitoflavone due to its strong antioxidant and radical scavenging properties [25]. However, as a diet compound, digitoflavone’s antioxidant function on gastrointestinal tract is not fully understood.

In the present work, we have investigated the action of digitoflavone to protect Caco-2 cells, a human cell line originating from gastrointestinal tract that retains many of the morphological and enzymatic features typical of normal human enterocytes, against oxidative stress and further *in vivo* study of its chemopreventive effect in AOM-DSS induced CRC model. Our results demonstrate for the first time that digitoflavone is able to attenuate oxidative injury in colonic cells by up-regulate the expression of the antioxidant defense enzymes via a mechanism that involved p38 MAPKs activation and Nrf2 translocation and further confirmed chemopreventive effect by free radical scavenging and inhibition of inflammation.

## Result

### Digitoflavone induced high levels of ARE-driven luciferase activities in Caco-2, HT-29, HepG2 and HEK-293 cells

A DNA fragment containing 8 copies of the ARE sequence (GTGACAAAGCACCC) were subcloned into the pGL3 vector. After transient transfection with the expression plasmid, different concentrations of digitoflavone were added to the cell culture and incubated for 8 hours and 24 hours respectively. Parallel cell viability assays revealed no obviously cytotoxic effects (>95% viability) for the digitoflavone treatment when the concentration of digitoflavone is lower than 10 μM in Caco-2, HepG2, HEK-293 cells and 5 μM in HT-29 cells (Figure 1F). 10 μM digitoflavone induced the highest level of luciferase activity after 8 hours exposure, about 5-fold increases of control (Figure 1B). Another human epithelial colorectal adenocarcinoma cell line HT-29 also showed that low concentrations (5 μM) of digitoflavone can increase the ARE-luciferase activity with no obviously cytotoxic effects (Figure 1C). To evaluate the ARE-driven luciferase activity of digitoflavone in other cell lines, HepG2 (Figure 1D) and HEK-293 (Figure 1E) cell lines were transient transfected with the pGL3-ARE-luciferase plasmid respectively and tested with 1–20 μM digitoflavone for 8 hours. All tested cell lines showed over 2-fold increases of the luciferase activity at 1–10 μM concentrations of digitoflavone. These result suggested that digitoflavone, at low concentrations (<10 μM), is a potent activator of the Nrf2/ARE antioxidant pathway.

### Digitoflavone stimulated the expression of the Nrf2-ARE-mediated antioxidant defense proteins in Caco-2 cells

**Figure 2:**
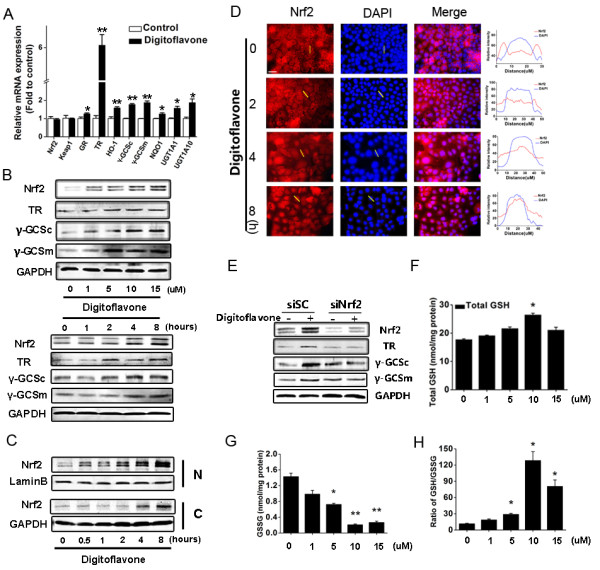
**Effects of digitoflavone on mRNA and protein levels of Nrf2-mediated phase II enzymes-antioxidant proteins, Nrf2 expression and nucleus accumulation in Caco-2 cells. (A)** Effects of digitoflavone on mRNA levels of the selected ARE genes. Caco-2 cells were treated with 10 μM digitoflavone for 8 hours and the mRNA levels of all selected genes were evaluated using RT-PCR. **(B)** Dose and time effect of digitoflavone. Caco-2 cells were treated with various concentrations (0–15 μM) of digitoflavone for 8 hours (top) and 10 μM digitoflavone for 0–8 hours (bottom). Nrf2, γ-GCSc, γ-GCSm and TR expression level were determined using Western blotting. **(C)** Time effect of Nrf2 nucleus accumulation. Caco-2 cells were treated with 10 μM digitoflavone for 0–8 hours. Total form Nrf2 in nucleus (N) and cytoplasm (C) were determined using Western blotting. **(D)** Immunofluorescence staining of Nrf2. Caco-2 cells were treated with 10 μM digitoflavone for 0–8 hours and then Nrf2 was labeled with immunofluorescence staining. Cells were counter-stained with DAPI for visualization of the nuclei. The left panel shows the relative signal-intensity profiles of Nrf2 and DAPI along the cross sections at the light yellow line. White scale bar indicates 25 μm. **(E)** Nrf2 silencing abrogates digitoflavone-induced cytoprotective protein expression. After 48 h transfection, the cells were treated with 10 μM digitoflavone for 8 hours and whole-cell lysates (TL) were then prepared. The protein levels of Nrf2, γ-GCSc, γ-GCSm and TR were analyzed. **(F-H)** Effects of digitoflavone on cellular total GSH, GSSG and the ratio of GSH/GSSG. Caco-2 cells were treated with various concentrations of digitoflavone for 8 hours, and the total GSH, GSSG and the GSH/GSSG ratio were analyzed according to the manufacturer’s instructions. The values represent the means ± SD of triplicate experiments. Significantly different (versus control group): *p<0.05, **p<0.01.

To verity whether activation of luciferase activity by digitoflavone in Caco-2 cells reflected the expression of the endogenous ARE-driven genes, the mRNA levels of GR, TR, HO-1, γ-GCSc, γ-GCSm, NQO1, and MRP2 were examined in the presence or absence of digitoflavone. In Caco-2 cells treated with 10 μM digitoflavone for 8 hours, the mRNA levels of GR, TR, HO-1, γ-GCSc, γ-GCSm, UGT1A1 and UGT1A10 increased 1.2-, 6.0-, 1.5, 1.7-, 1.8-, 1.5, 1.8-fold, respectively (Figure 2A). Similarly, evaluation of the Nrf2-mediated antioxidant enzymes, such as γ-GCSc and TR by Western blotting showed that exposure of Caco-2 cells to 1–15 μM digitoflavone strongly induced γ-GCSc, γ-GCSm and TR protein expression in a dose and time-dependent manner (Figure 2).

### Digitoflavone induced Nrf2 protein expression and nuclear translocation

Previous studies described that under normal conditions, Keap1 sequestered Nrf2 in the cytoplasm and that translocation of Nrf2 into the nucleus is essential for the transactivation of various targeted genes [26,27]. Therefore, to further investigate effects of digitoflavone one the Nrf2/ARE activation, we examined the protein expression and subcellur location of Nrf2 in Caco-2 cells after digitoflavone treatment. As show in the Figure 2B, Western blot analysis demonstrated a significant increase of Nrf2 protein expression after digitoflavone treatment in dose- and time-dependent manner. Western blot analysis of the nuclear fraction (Figure 2C) and Immunofluorescence analyses (Figure 2D) showed Nrf2 accumulation in the nucleus of Caco-2 cells after digitoflavone treatment.To confirm the requirement of Nrf2 in the digitoflavone-induced antioxidant activities, we transfected the Caco-2 cells with Nrf2 target siRNA before digitoflavone treatment. As show in Figure 2E, silencing Nrf2 expression significantly inhibited the digitoflavone-induced γ-GCSc, γ-GCSm and TR up-regulation, suggesting that digitoflavone induced antioxidant activities in an Nrf2/ARE-dependent manner.We also investigated changes in GSH content in Caco-2 cells after incubation in varying concentrations of digitoflavone for 8 h. Digitoflavone increased GSH content and decreased the level of GSSG in a dose-dependent manner, which resulted in a dose-dependent increase in the ratio of GSH/GSSG (Figure 2F-H). This result is consistent with increased levels of γ-GCSc and γ-GCSm mRNAs, which encode the rate-limiting enzymes in GSH synthesis, in Caco-2 cells.

### Digitoflavone exhibited cytoprotective effects against H_2_O_2_-induced oxidative stress in Caco-2 cells

**Figure 3:**
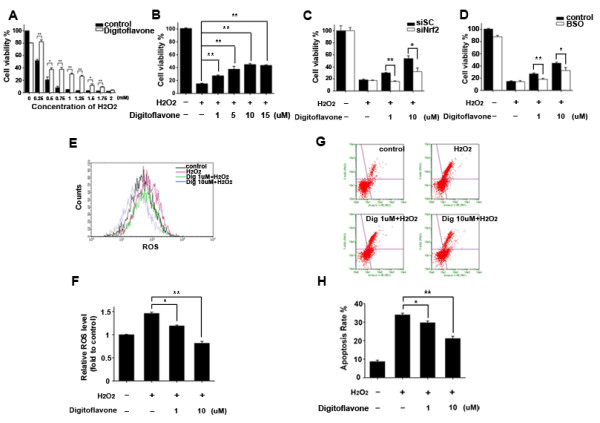
**Effects of digitoflavone on oxidative stress-induced cell injury in Caco-2 cells. (A)** The anti-oxidative ability of digitoflavone. Caco-2 cells were pretreated with 10 μM digitoflavone for 4 hours then espoused to various concentration of H_2_O_2_ for an additional 24 h. Cell viability was determined using MTT assay. **(B)** Dose-dependent anti-oxidative ability of digitoflavone. Caco-2 cells were pretreated with 1–15 μM digitoflavone for 4 hours then espoused to 500 μM H_2_O_2_ for an additional 24 h. Cell viability was determined using MTT assay. **(C)** Nrf2 silencing partly abrogates digitoflavone-induced cytoprotective effect. After 48 h transfection, the cells were treated with 10 μM digitoflavone for 4 hours and followed an oxidative stress of 500 μM H_2_O_2_ for 24 hours, Cell viability was determined using MTT assay. **(D)** GSH synthetise inhibitor-BSO partly abrogates digitoflavone-induced cytoprotective effect. Caco-2 cells were pretreated with 10 μM BSO for 2 hours, then cells were treated with 10 μM digitoflavone for an additional 4 hours, anti-oxidative ability was tested with 500 μM H_2_O_2_ for an additional 24 h using MTT assay. **(E)** Flow cytometry analysis of the intracellular ROS. Caco-2 cells were treated with various concentrations of digitoflavone for 4 hours before espoused to 500 μM H_2_O_2_ for an additional 4 h. Intracellular ROS levels were measured using DCF fluorescence. **(F)** Statistical analysis of the flow cytometry date. **(G)** Flow cytometry analysis of the apoptotic rate. Caco-2 cells were treated with various concentrations of digitoflavone for 4 hours before espoused to 500 μM H_2_O_2_ for an additional 6 h. cells were stained with FITC-Annexin V-PI, flow cytometry measured the apoptotic rate. **(H)** Statistical analysis of the apoptotic rate. The values represent the means ± SD of triplicate experiments. Significantly different: *p<0.05, **p<0.01.

Nrf2 is a key component in protection against carcinogenesis and oxidative stress [26,28,29]. Previous reports have suggested that oxidative stress plays an important role in tumor promotion [30,31]. H_2_O_2_ may induce self-generation of free radicals known as the ROS-induced ROS release at the mitochondrial level [32], which has been widely used as a model of exogenous oxidative stress. In this study, we validated if antioxidant activities induced by digitoflavone can actually protect against H_2_O_2_-induced damage in Caco-2 cells. The protective effects of digitoflavone against the H_2_O_2_-induced cytotoxicity were detected by MTT assay. As show in Figure 3A and B, pretreatment of digitoflavone for 4 h exhibited dose-dependent protective effects in the H_2_O_2_-damage model and the Nrf2 target siRNA transfection group (Figure 3C), while the GSH synthesis inhibitor BSO partially abolished the digitoflavone-induced protective effect (Figure 3D).

Intracellular ROS levels affect cell viability and high ROS levels can cause cellular damage. Using flow cytometry analysis, we examined the effects of digitoflavone on intracellular ROS levels. As shown in Figure 3E and F, H_2_O_2_ treatment led to a significant increase in ROS levels. Statistical analysis showed that digitoflavone reduced the H_2_O_2_-induced intracellular ROS level in a dose-dependent manner. We further confirmed the anti-apoptotic effects of digitoflavone through the quantitative analysis of FITC-Annexin V/PI staining by flow cytometry. In the normal control group, the percentage of apoptotic cells was 8.7%. The percentage of apoptotic cells increased up to 33.9% in the H_2_O_2_ model group (Figure 3G and H). The protective effects of digitoflavone against cell apoptosis was concentration dependent (apoptosis rate 29.7 ± 0.88% and 21.2 ± 1.18% for 1 and 10 μM digitoflavone treatment respectively).

### Role of p38 MAPK in the digitoflavone-induced Nrf2-ARE activation in Caco-2 cells

**Figure 4:**
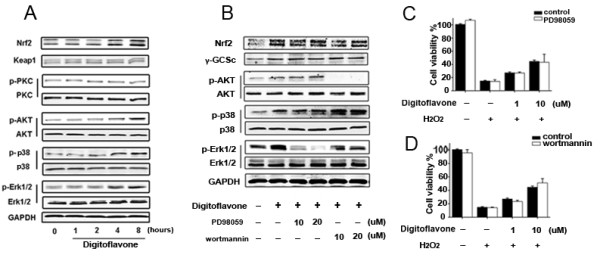
**Effects of signaling inhibitors on digitoflavone-induced Nrf2 and γ-GCSc expression in Caco-2 cells. (A)** Possible mechanism of digitoflavone-induced Nrf2 activation. Caco-2 cells were treated with 10 μM digitoflavone for 0, 1, 2, 4, 8 hours, Keap1 expression level and Nrf2 upstream kinases were determined using Western blotting and appropriate specific antibodies. **(B)** Role of Erk1/2 and PI3K/AKT in digitoflavone-induced Nrf2 activation. Caco-2 cells were pretreated with various concentration of PD98059 (MEK1/2 inhibitor), wortmannin (for PI3K inhibitor) for 2 hours respectively, then cells were treated with 10 μM digitoflavone for an additional 8 hours, Nrf2 and γ-GCSc expression were determined using Western blotting. **(C-D)** Caco-2 cells were pretreated with 10 μM PD98059 or 10 μM wortmannin for 2 hours respectively, then cells were treated with 10 μM digitoflavone for an additional 4 hours, anti-oxidative ability was tested with 500 μM H_2_O_2_ for an additional 24 h using MTT assay. The values represent the means ± SD of triplicate experiments. Significantly different: *p<0.05, **p<0.01.

**Figure 5:**
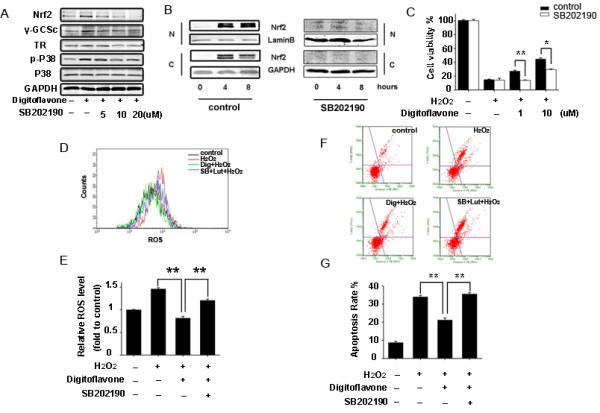
**Digitoflavone activates the Nrf2/ARE axis and induces cytoprotective effect via the p38 MAPK pathway. (A)** Effects of p38 signaling inhibitoγ-SB202190 on digitoflavone-induced Nrf2 expression. Caco-2 cells were pretreated with various concentration of p38 signaling inhibitors-SB202190 for 2 hours then treated with 10 μM digitoflavone for an additional 8 hours. Nrf2, γ-GCSc and TR protein expression was determined using Western blot analysis. **(B)** Effects of p38 signaling inhibitor-SB202190 on digitoflavone-induced Nrf2 translocation. Caco-2 cells were pretreated with 10 μM SB202190 for 2 hours then treated with 10 μM digitoflavone for 0, 4, 8 hours. Nrf2 in nucleus (N), cytoplasm (C) were determined using Western blotting and appropriate specific antibodies. Lamin B and GAPDH were used as internal controls for nuclear and cytoplasmic extracts, respectively. **(C)** Cell viability analysis. Caco-2 cells were preincubated with or without 10 μM SB202190 then treated with various concentrations of digitoflavone for 4 hours before espoused to 500 μM H_2_O_2_ for an additional 24 hours. Cell viability was determined using MTT assay. **(D)** Flow cytometry analysis of the intracellular ROS. Caco-2 cells were preincubated with or without 10 μM SB202190 then treated with various concentrations of digitoflavone for 4 hours before espoused to 500 μM H_2_O_2_ for an additional 4 hours. Intracellular ROS levels were measured using DCF fluorescence. **(E)** Statistical analysis of the flow cytometry date. **(F)** Flow cytometry analysis of the apoptotic rate. Caco-2 cells were preincubated with or without 10 μM SB202190 then treated with various concentrations of digitoflavone for 4 hours before espoused to 500 μM H_2_O_2_ for an additional 6 hours. cells were stained with FITC-Annexin V-PI, flow cytometry measured the apoptotic rate. **(G)** Statistical analysis of the apoptotic rate. The values represent the means ± SD of triplicate experiments. Significantly different: *p<0.05, **p<0.01.

Under normal conditions, the interaction of Nrf2 with the Kelch-like ECH-associated protein 1 (Keap1) traps Nrf2 in the cytosol, leading to a rapid degradation of the cytosolic Nrf2 by the 26S proteasome, via the Cullin3-based E3-ligase ubiquitination complex [26]. A number of studies have shown that several signaling pathways, including PI3K [33,34], MAPK [35,36], and PKC [37,38], are involved in the induction of Nrf2/ARE-driven gene expression. To elucidate the signal transduction pathways leading to the activation of Nrf2 and the induction of antioxidants expression in the digitoflavone-treated cells, we examined the effects of digitoflavone on the expression of Keap1 and the phosphorylation of PKC, AKT, ERK1/2, and p38 MAPK. Upon digitoflavone treatment, time-dependent increases in the phosphorylation of AKT, ERK1/2, and p38 MAPK were observed (Figure 4A). To determine whether such activations of AKT, ERK1/2, and p38 MAPK contribute to the digitoflavone-induced Nrf2 activation, several kinase inhibitors, including wortmannin (for PI3K), PD98059 (for MEK1/2), and SB202190 (for p-p38 MAPK), were employed. As show in Figure 4B-D, inhibition of the phosphorylation of AKT and ERK1/2 did not decrease the digitoflavone-induced Nrf2 activation. However, the p38 MAPK inhibitor SB202190 significantly inhibited the digitoflavone-induced Nrf2 activation (Figure 5A) and nuclear accumulation (Figure 5B). To determine whether such activation of p38 MAPK contribute to the digitoflavone-mediated protections against the cytotoxic effects of H_2_O_2_, the Caco-2 cells were pre-incubated with SB202190 for 2 hours before the 4 hours digitoflavone treatment, Cells were then challenged with 500 μM H_2_O_2_ for additional 24 h for MTT assay, 4 h for ROS detection, and 6 h for apoptosis detection, respectively. As show in Figure 5C, SB202190 eliminated the protective effects of digitoflavone. SB202190 also reversed the digitoflavone antioxidant activity (Figure 5D and E). Further, the anti-apoptosis ability of digitoflavone was also abolished by SB202190 (Figure 5F and G).

### The chemopreventive effect of digitoflavone on tumor progression in mice

**Figure 6:**
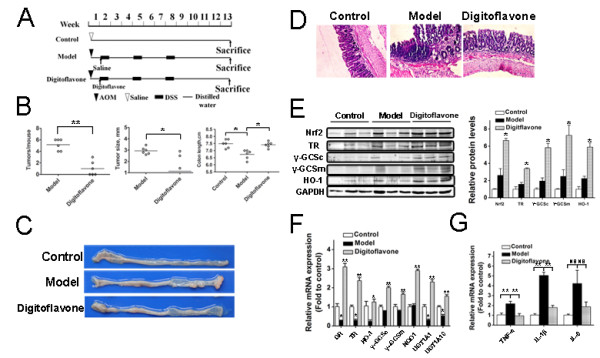
**The effects of digitoflavone on colon carcinogenesis. (A)** Schematic overview of digitoflavone administration. Colons were removed at week 13 after the mice were administered digitoflavone or a vehicle control between week 2 and 13. **(B)** The tumor numbers, sizes and colon length were determined macroscopically. The bars represent the median of each group. Each symbol represents the tumor numbers and colon length of each animal or the average size of the tumors of each animal. Significantly different: *p<0.05, **p<0.01. **(C)** Macroscopic evaluation of the tumors. Colons were removed on week 13 from mice, treated with digitoflavone or with vehicle. Representative results from 6 independent animals are shown here. Original magnification, ×6. **(D)** Colons were processed for hematoxylin and eosin staining and representative results from 6 independent animals are shown here. Original magnification, × 40. **(E)** Immunoblotting analysis with anti–Nrf2, TR, γ-GCSc, γ-GCSm and HO-1 antibodies was performed on cell lysates from colon tissues as described in Methods. Representative results from 3 independent experiments are shown here (left panel), the intensity of bands from replicate immunoblots was quantified and plotted (right panel, bar graphs). Significantly different (versus control group): *p<0.05, **p<0.01. **(F-G)** Quantitative RT-PCR analysis for GR, TR, HO-1, γ-GCSc, γ-GCSm, NQO-1, UGT1A1, UGT1A10, TNF-a, IL-1βand IL-6 was performed on total RNAs extracted from the colons. All genes’ mRNA levels were normalized to the levels of GAPDH mRNA. Significantly different (versus control group): *p<0.05, **p<0.01 and NS, not significant.

We further explored chemopreventive effects of digitoflavone on tumor progression by administering it to mice from week 2 to day 13, after the AOM and 3 cycles of DSS treatments (Figure 6A). Compared with the AMO group, digitoflavone treatment reduced the numbers and size of macroscopical tumors remarkably and the shorted colon length was resvered by digitoflavone when compared with AOM group, also less loss of crypts was observed in mice with digitoflavone treatment (Figure 6B-D). Next, activation of Nrf2 and its downstream targets were assessed to demonstrate that the beneficial effect of digitoflavone against tumor progression is attributed to activation of the Nrf2 pathway. Protein expression of Nrf2 and Nrf2 downstream targets TR, γ-GCSc and γ-GCSm and mRNA expression of GR, TR, HO-1, γ-GCSc, γ-GCSm, NQO-1, UGT1A1 and UGT1A10 were slightly changed in AOM group, indicating induction of the Nrf2 pathway by colon oxidative stress. As expected, treatment with digitoflavone markedly increased the protein levels of Nrf2, TR, γ-GCSc, γ-GCSm and HO-1 and mRNA levels of GR, TR, HO-1, γ-GCSc, γ-GCSm, NQO-1, UGT1A1 and UGT1A10 (Figure 6E and F). The mRNA Levels of colonic inflammatory cytokines TNF-a, IL-1β and IL-6 were increased in AOM group, and digitoflavone reduced TNF-a, IL-1β and IL-6 mRNA Levels when compared with AOM group (Figure 6G).

## Discussion

The intestinal epithelium sits at the interface between an organism and its luminal environment, and as such is prone to oxidative damage induced by luminal oxidants. The intestinal epithelial cells, as a barrier between an organism and its intestinal contents, are the first line of defense against frequent exposure to xenobiotics containing chemical toxicants [39]. In the biological defense process, intestinal epithelia express detoxification enzymes that play important roles in metabolism, detoxification, and exclusion of the xenobiotics [40]. Oxidative stress is associated with mucosal erosions and has a causative role in a variety of gastrointestinal diseases such as Crohn’s disease and ulcerative colitis [41,42]. In particular, dietary pro-oxidants may alter the redox status of intestinal cells and provoke inflammatory bowel disease and colon cancer. Epidemiological studies have related a diet rich in fruits and vegetables to the prevention of chronic degenerative diseases linked to oxidative stresses [43]. The antioxidant and chemoprotective properties of food flavonoids or polyphenolic extracts have been widely reported in cultured cells [43], animal models [44], and humans [45,46].

There is a substantial body of scientific literatures that supports a positive role of flavonoids on health [13]. The mechanisms by which the specific flavonoids exert these benefits are under intense investigation. Digitoflavone is a common dietary flavonoid that can be found in a large number of plants and foods and it has been found to possess anti-oxidant, anti-inflammatory/anti-allergic, anti-tumorigenic, and radical action [47,48]. In this study, we demonstrated that the flavones digitoflavone inhibited H_2_O_2_-induced oxidative stress and that this suppression was likely associated with the up-regulation of γ-GCSc and γ-GCSm expression through the p38/Nrf2 pathway.

The human colon carcinoma cell line Caco-2 and its derivatives have been widely used in studies on molecular effects of and inter-actions with xenobiotics. The cell line undergoes differentiation during culture, which results in an ileum cell like model system, as well as a model system for cells of the small intestine [49,50]. Using the Caco-2 cells, we explored the potential molecular mechanisms underlying the chemopreventive and antioxidant effects of digitoflavone, focusing on ARE activation. We found that digitoflavone acts as an ARE inducer not only in colon cells Caco-2 s (Figure 1B) and HT-29 (Figure 1C), but also in many other types of cells (Figure 1D-E). Numerous studies have suggested that ARE sequences are involved in regulating the expression of a wide array of antioxidant and detoxifying genes [51], and Nrf2 serves as a master regulator of the ARE-driven cellular defense system against oxidative stresses.

Under normal conditions, Nrf2 is sequestered by Keap1, a substrate adaptor, which helps Cullin 3 ubiquitinate Nrf2 in the cytoplasm, and ARE activation signals (i.e., protein kinase pathway and redox-active components) disrupt the Nrf2/Keap1 complex, leading to phosphorylation and nuclear translocation of Nrf2. Nrf2 then heterodimerizes with small Maf and binds to ARE, eventually resulting in transcriptional activation of the ARE-mediated metabolizing/detoxifying and antioxidant genes [9]. We report in this study that digitoflavone strongly induced Nrf2 protein expression and nucleus accumulation (Figure 2B-D). The rapid accumulation of Nrf2 in the nucleus in response to digitoflavone is consistent with reported results with other Nrf2 activators, such as PEITC [36] and celecoxib [34], and with the Nrf2 degradation inhibitors such as eckol [52].

The Nrf2/ARE pathway activates approximately 100 cytoprotective genes [14]. In this study, digitoflavone elevated the mRNA and protein levels of several ARE-mediated antioxidant/detoxifying genes in Caco-2 cells (Figure 2A and B). Knockdown of Nrf2 by Nrf2-targeted siRNA markedly suppressed the digitoflavone-induced γ-GCSc, γ-GCSm expression (Figure 2D), suggesting that digitoflavone up-regulates Nrf2-dependent activation of the ARE-regulated genes. Nrf2 controls the expression of γ-GCSc and γ-GCSm, which together catalyze the rate-limiting step in GSH biosynthesis [53]. Involvement of GSH in the digitoflavone-induced cytoprotection against oxidative injury could not be excluded, because increasing GSH levels would be expected to reduce ROS levels and antagonize the ROS-induced cell death [53]. In this study, treatment of cells with digitoflavone resulted in decreased H_2_O_2_-induced oxidative stress (Figure 3E and F), and cell death (Figure 3G and H).

Activation of Nrf2 involves regulation of protein kinases, which may induce Nrf2 phosphorylation and nuclear translocation [54-57]. The MAPK cascade, PI3K/AKT, and PKC signaling pathways have been reported to influence the Nrf2/ARE pathway [57]. For example, phosphorylation of Nrf2 by PKC promotes its release from Keap1 [6] and inhibition of PI3K attenuates the nuclear translocation of Nrf2 and transcription of ARE-mediated genes [58]. To identify which signal cascade controlled activation of Nrf2 by digitoflavone, we examined the effects of PI3K inhibitor, ERK1/2 inhibitor, and p38 MAPK inhibitor on the digitoflavone-induced Nrf2 up-regulation. Our results demonstrated that PI3K/AKT and ERK1/2 are not involved in the digitoflavone-induced activation of the Nrf2/ARE pathway because their inhibitor had no effect on enhanced digitoflavone-induced Nrf2 up-regulation (Figure 4B-D). On the contrary, inhibition of p38 MAPK by SB202190 leads to decrease of the digitoflavone-induced Nrf2 up-regulation, indicating that the digitoflavone-induced Nrf2 activation is dependent on the activation of p38 MAPKs. Inhibition of p38 also abrogated the digitoflavone-induced translocation of Nrf2 to nucleus and the antioxidant defense effect, demonstrating that the crucial role of p38 in the Nrf2-dependent activation of ARE and suggesting that Nrf2 is a downstream effector of p38 kinase in response to digitoflavone treatment.

**Figure 7:**
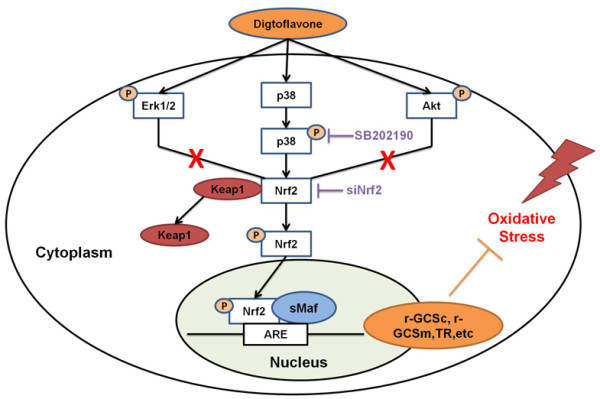
**A proposed pathway for digitoflavone-induced Nrf2/ARE-mediated cytoprotective proteins via activation of p38 MAPK signaling.** Up-regulation of γ-GCSc, γ-GCSm explains the cytoprotective effects against oxidative stress in Caco-2 cells.

*In vivo* experiment we study the chemopreventive role of digitoflavone in AOM-DSS induced colorectal cancer model. Digitoflavone was post-treated after the initiation of stage of colorectal cancer. Compared with AOM group, digitoflavone group shown lower cancer incidents (50% compare with AMO group 100%), reduced numbers and size of macroscopical tumors and recovered colon length (Figure 6B-C). General histological observation found that digitoflavone retained a better colonic histoarchitecture with less loss of crypts (Figure 6D). Further protein and mRNA level Analysis indicated the chemopreventive role of digitoflavone may through the activation of Nrf2 and inhibition of inflammation (Figure 6E-G).In summary, our study demonstrates for the first time that digitoflavone improved the intestinal antioxidant potential through the induction of the main detoxification enzyme γ-GCSc and γ-GCSm by a mechanism in which activation of p38 MAPK plays an essential role (Figure 7). In addition, digitoflavone was identified as a potent inducer of Nrf2 expression and translocation providing a support for the involvement of this transcription factor in the induction of γ-GCSc and γ-GCSm. The results of the present study add further evidence of the molecular mechanisms that allow digitoflavone to exert protective effects and reaffirm its potential role as a chemopreventive agent in colorectal carcinogenesis.

## Material and method

### Material

AOM, DSS (MW 36,000–50,000), digitoflavone, SB202190, DCFH-DA [2′, 7′-Dichlorofluorescin diacetate], Trypsin, MTT [3-(4, 5-dimethylthiazol-2-yl)-2, 5-diphenyltetrazoliμM bromide], BSO [L-Buthionine-sulfoximine ], DNase-free RNase and SB202190 were obtained from Sigma-aldrich, USA. Digitoflavone was dissolved in dimethyl sulfoxide (DMSO) and was used in all experiments. Maxima**®** SYBR Green/ROX qPCR Master Mix (2×) and Maxima**®** First Strand cDNA Synthesis Kit were purchased from Fermentas life science (Fermentas, MBI). PD98059, Wortmannin, Lysis buffer was purchased from Beyotime, China. Primary antibodies (Nrf2 for mouse, γ-GCSc, γ-GCSm, TR, HO-1, Keap1, PKC, Lamin B) were obtained from Santa Cruz Biotechnology, CA, USA. Rabbit anti-Nrf2 (for human) was purchased from Abcam, USA. Primary antibodies (p-PKC, ERK1/2, p-ERK1/2, p-p38, p38, AKT and p-AKT) were purchased from Cell Signaling Technology, MA, USA. Goat anti-rabbit IgG and goat anti-mouse IgG antibodies were purchased from LI-COR, Lincoln, NE, USA. Rabbit anti-Goat IgG was obtained from KPL, Gaithersbhrg, MD, USA. Monoclonal mouse anti-glyceraldehyde-3-phosphate dehydrogease (GAPDH) was obtained from KangChen, China.

### Cell lines and cell culture

Human epithelial colorectal adenocarcinoma cell line Caco-2, Human colon adenocarcinoma grade II cell line HT-29, human liver carcinoma cell line HepG2 ,Human Embryonic Kidney 293 cell HEK-293 were purchased from Cell Bank of Shanghai Institute of Biochemistry and Cell Biology. Cells were cultured in DMEM-medium (for HepG2, HEK-293 cells), MEM-medium (for Caco-2 cells), McCOY’s 5A(for HT-29) supplemented with 10% fetal bovine serum (FBS), 100 U/ml penicillin and 100 μg/ml streptomycin (all available from Invitrogen, Grand Island, NY, USA). All cultures were maintained in a humidified environment with 5% CO^2^ at 37°C.

### Transient transfection and analysis of luciferase reporter gene activity

We used the luciferase reporter assay to investigate the Nrf2-mediated transcriptional activity of Nrf2. Firstly, 8 copies of antioxidant-responsive element (ARE)–luciferase reporter plasmids were generated using the pGL3 promoter vector (Promega UK). After the plasmids were generated, the DNA sequence of the inserts was verified. The Dual-Luciferase Reporter Assay System (Promega, UK) was used to determine reporter gene activity in transiently transfected cells. Transient transfection was performed in 96-well plates at a cell density of 50% ~ 70% confluence per well. Then the 8 × ARE pGL3 plasmid were co-transfected with the pRL-TK plasmid(transfection ratio of 8 × ARE pGL3:pRL-TK is 10:1), encoding Renilla luciferase as an internal control for transfection efficiency for 24 h using Lipofectamine 2000 (Invitrogen, Carlsbad, CA, USA) according to the manufacturer’s instructions. After transfection, cells were treated with test samples for indicated time, and then cell lysates were prepared for assessment of luciferase activity. Fire fly and Renilla luciferase activities were measured using a luminometer (Centro XS3 LB960, Berthold, Germany) according to the manufacturer’s instructions. Relative fire fly luciferase activity was normalized to Renilla luciferase activity and activity was expressed as fold induction after treatment with compounds compared with vehicle control (DMSO).

### Cell viability assay

Cell viability was determined using the MTT assay. Briefly, cells in logarithmic phase were seeded at the density of 70 ~ 80% confluence per well in 96-well plates at 37°C with 5% CO^2^ for overnight incubation and treated with appropriate concentrations of test samples for the indicated times. After treatment, 10 μl of 5 mg/ml MTT was added and the cells were incubated for 4 h at 37°C. The supernatant was discarded and 100 μl of DMSO was added to each well. The mixture was shaken on a mini shaker at room temperature for 10 min and the spectrophotometric absorbance was measured by Multiskan Spectrum Microplate Reader (Thermo, USA) at 570 nm and 630 nm (absorbance 570 nm, reference 630 nm). Triplicate experiments were performed in a parallel manner for each concentration point and the results were presented as mean ± SD. The net *A*_570nm_-*A*_630nm_ was taken as the index of cell viability. The net absorbance from the wells of cells cultured with DMSO was taken as the 100% viability value. The percent viability of the treated cells was calculated by the formula: % viability = (*A*_570nm_-*A*_630nm_) _treated_/(*A*_570nm_-*A*_630nm_) _control_ × 100%.

### SDS –PAGE and Western blot analysis

Caco-2 cells were cultured in MEM and then treated with test samples for indicated time. Proteins were isolated by lysis buffer (Beyotime, China) and measured using the Nanodrop 1000 Spectrophotometer (Thermo, USA). Protein samples were separated on 10% SDS-polyacrylamide gels (SDS-PAGE) and transferred onto the PVDF membranes (Millipore, USA). After blocked with 1% BSA in TBST for 2 h, membranes were incubated with primary antibodies overnight at 4°C. Blots were washed and incubated with secondary antibodies for 1 h at room temperature. Membranes were again washed three times with TBST and were scanned with an Odyssey infrared fluorescent scanner (LI-COR) and analyzed with Odyssey software version 3.

### Determination of cellular reduced glutathione (GSH) content

Caco-2 cells were treated with various concentrations of digitoflavone (1–15 μM) or vehicle control (DMSO). After 8 h incubation, the cellular GSH and GSSG were quantified using GSH/GSSG-Glo Assay kit (Promega) according to the manufacturer’s protocol. GSH and GSSG levels were normalized to protein concentrations and the GSH/GSSG ratio was calculated.

### Immunofluorescence staining

Cells in logarithmic phase were seeded in logarithmic phase were seeded at the density of 70 ~ 80% confluence per well into 24-well chamber slides. After treatment with test samples for the indicated times, cells were fixed with cold 4% (w/v) paraformaldehyde for 20 min, rehydrated in PBS for 15 min, and permeabilized in 0.1% (w/v) TritonX-100 at room temperature for 10 min. After being washed with PBS, the cells were blocked unspecific fluorescence with 3%BSA for 1 hour and then incubated with primary antibody at 4°C overnight followed by Texas Red-conjugated secondary antibody for 1 h at room temperature. The images of Nrf2 with Texas Red staining were captured using a fluorescence microscope.

### Preparation of nuclear extract proteins

Nuclear extract protein was prepared according manufactory’s instruction (Thermo, USA). Briefly, after treatment with digitoflavone for indicated times, Caco-2 cells were harvested, washed with PBS, centrifuged, and resuspended in ice-cold buffer CERI. After 10 min of incubation on ice, cells were added with ice-cold CERII and centrifuged again, the supernatant (cytoplasmic extract) was immediately transferred to a clean pre-chilled tube. The insoluble (pellet) fraction was resuspended with NER, and vortex for 15 seconds every 10 min for a total 40 min. The tube was centrifuged and the supernatant (nuclear extract) was immediately transferred to a clean pre-chilled tube. The cytoplasmic and nuclear extract protein was stored at −80°C until use. For Western blot analysis, LaminB and GAPDH were used as internal controls for nuclear and cytoplasmic extracts, respectively.

### Real-time reverse transcription –polymerase chain reaction (RT-PCR)

Caco-2 cells were treated with different concentrations of digitoflavone for indicated times, then treated cells were washed with PBS, total RNA was extracted from the treated cells using trizol reagent (Invitrogen, Carlsbad, CA) and then RNA was converted to cDNA by reverse transcriptase (Thermo, USA) according to the manufacturer’s instruction. Primers used for the reactions were purchased from Genscript and the sequences were listed in Table 1. Real-time qPCR analysis for mRNA expression was performed using SYBR Green probes and an ABI 7500. ALL genes’ mRNA expression was normalized against GAPDH expression.

**Table 1:** Primers used for real-time RT-PCR

**Primer**	**Sequences**
hNrf2	Forward primer,5′- CATCCAGTCAGAAACCAGTGG
	Reverse primer ,5′- GCAGTCATCAAAGTACAAAGCAT
hKeap1	Forward primer, 5′-CCTTCAGCTACACCCTGGAG
	Reverse primer, 5′-CATGACCTTGGGGTGGATAC
hGR	Forward primer, 5′-CACGGA GGAGCTGGAGAAC
	Reverse primer, 5′- CGACAAAGTCTTTTTAACCTCCTT
hTR	Forward primer, 5′-CAGACG GGGAGGCTTTTC
	Reverse primer, 5′- CCGAGAGCGTTCCTTTCA
hHO-1	Forward primer, 5′- TCCTGGCTCAGCCTCAAATG;
	Reverse primer, 5′- CGTTAAACACCTCCCTCCCC
hr-GCSc	Forward primer, 5′-CTGTTGCAGGAAGGCATTGAT
	Reverse primer, 5′- TTCAAACAGTGTCAGTGGGTCTCT
hr-GCSm	Forward primer, 5′- GGCACAGGTAAAACCAAATAGTAAC
	Reverse primer, 5′- CAAATTGTTTAGCAAATGCAGTCA
hNQO1	Forward primer, 5′- GGGATCCACGGGGACATGAATG
	Reverse primer, 5′- ATTTGAATTCGGGCGTCTGCTG
hUGT1A1	Forward primer, 5′-TAAGTGGCTACCCCAAAACG
	Reverse primer, 5′- TCCAGCTCCCTTAGTCTCCA
hUGT1A10	Forward primer, 5′- CGTGTTCTGGGTGGAGTTTG
	Reverse primer, 5′- TTTTCCCCAAGCATTTCCGG
hGAPDH	Forward primer, 5′- TGCACCACCAACTGCTTAGC
	Reverse primer, 5′- GGCATGGACTGTGGTCATGAG
mGR	Forward primer, 5′- AGTGCACTCGGAATTCATGC
	Reverse primer, 5′- CAATCAGGATGTGTGGAGCG
mTR	Forward primer, 5′- ATCCACAAACAGCGAGGAGA
	Reverse primer, 5′- TTGGTCTGCTCTTCATCCGT
mHO-1	Forward primer, 5′- GCCACCAAGGAGGTACACAT
	Reverse primer, 5′- GCTTGTTGCGCTCTATCTCC
mr-GCSc	Forward primer, 5′- GGCCACTATCTGCCCAATTG
	Reverse primer, 5′- TGTTCTTCAGAGGCTCCAGG
mr-GCSm	Forward primer, 5′- GGAGGGGCTCTTAACTCCAG
	Reverse primer, 5′- CTCAACACAGTGCCGAACAA
mNQO1	Forward primer, 5′- ACAGGTGAGCTGAAGGACTC
	Reverse primer, 5′- GTTGTCGTACATGGCAGCAT
mUGT1A1	Forward primer, 5′- GGAGGCTGTTAGTGTTCCCT
	Reverse primer, 5′- CCGTCCAAGTTCCACCAAAG
mUGT1A10	Forward primer, 5′- GACTCGGGCATTCATCACAC
	Reverse primer, 5′- GCGCATGATGTTCTCCTTGT
mTNF-a	Forward primer, 5′- GACCCCTTTACTCTGACCCC
	Reverse primer, 5′- AGGCTCCAGTGAATTCGGAA
mIL-1b	Forward primer, 5′- ACTCATTGTGGCTGTGGAGA
	Reverse primer, 5′- TTGTTCATCTCGGAGCCTGT
mIL-6	Forward primer, 5′- CTGCAAGAGACTTCCATCCAGTT
	Reverse primer, 5′- GAAGTAGGGAAGGCCGTGG
mGAPDH	Forward primer, 5′- CTCCCACTCTTCCACCTTCG
	Reverse primer, 5′- CCACCACCCTGTTGCTGTAG

### Measurement of ROS

The production of cellular ROS, mainly H_2_O_2_, was detected using the DCFH-DA fluorescence assay. Briefly, cells were seeded in 24-well plates at the density of 70 ~ 80% confluence per well for overnight incubation. After treatment with appropriate concentrations of test samples, cells were harvested, placed into 1.5 mL round-bottom polystyrene tubes, and washed with PBS twice. Subsequently, the cells were centrifuged for 5 min at 400 × g at room temperature, and the supernate was discarded. The cells were resuspended in 500 μL ROS detection solution, stained in the dark at 37°C for 30 min, and analyzed by FACScan laser flow cytometer (Guava easycyteHT, Millipore, CA).

### Flow cytometric detection of apoptosis

Caco-2 cells in logarithmic phase at were treated with test samples for indicated time. Then they were harvested, washed and resuspended with PBS. Apoptotic cells were determined with an FITC Annexin V Apoptosis Detection Kit (BD Biosciences, USA) according to the manufacturer’s protocol. Briefly, the cells were washed and subsequently incubated for 15 min at room temperature in the dark in 100 ul of 1 × binding buffer containing 5ul of Annexin V-FITC and 5 ul of PI. Afterward, apoptosis was analyzed by FACScan laser flow cytometer (Guava easycyteHT, Millipore, CA).

### RNA interference study

Nrf2-specific short interfering RNA (siRNA) and scramble control siRNA were obtained from RIBOBIO (GuangZhou, China). Transfection was performed using LipofectAMINE 2000, according to the manufacturer’s protocol, with Nrf2-specific siRNA SMARTpool L-003755-00-0050; human NFE2L2 (NCBI Accession No. NM006164); target sequences including TCCCGTTTGTAGATGACAA, GAGAAAGAATTGCCTGTAA and GCAACAGGACATTGAGCAA. Briefly, cells were transfected with 10 nmol/L siRNAs directed against Nrf2 and non-targeting scramble control siRNA for 48 h, followed by treatment with the test samples for the indicated times. The cells were harvested and the protein status, MTT test and flow cytometry analysis.

### Animal experiments

All animal experiments were conducted in accordance with the NIH Guidelines for the Care and Use of Laboratory Animals. Pathogen-free 8- to 12-week old C57BL/6 male mice were housed in a temperature-controlled room (22°C) with a controlled 12-h light/dark cycle. The mice were given free access to diet and water during the course of experiments. They were allowed to adapt to the Experimental Animal Laboratory for 1 week before beginning the experiment. Mice were injected intraperitoneally with 10 mg/kg body weight of AOM dissolved in physiological saline. One week later, 2% DSS was given in the drinking water over 7 days, followed by 14 days of regular water. This cycle was repeated a total of 3 times. Body weight was measured every week, and the animals were sacrificed at week 13 for macroscopical inspection, histological analysis, and total RNA and protein extraction. In digitoflavone group, digitoflavone at 50 mg/kg dose suspended in 0.5% carboxymethyl cellulose (CMC) was given as gavage to mice and mice of control group and AOM group were given 0.2 mL 0.5% CMC solution every day from week 2 to week 13 (Figure 6A).

### Statistical analysis

Results are expressed as mean ± SD. Statistical tests were performed using SPSS 15.0. Unpaired Student t tests were used to compare the means of two groups. For multiple comparisons between groups, a one-way ANOVA was performed to detect statistical differences. Differences within the ANOVA were determined using a Tukey’s post-hoc test. P value of less than 0.05 was considered to be statistically significant.

## Abbreviations

ABCC: ATP-binding cassette, subfamily C member; AOM: Azoxymethane; AKR1C: Aldo-ketoreductase family member; ARE: Antioxidant-response element; DCFH-DA: 2′ ,7′ -chloro fluorescein diacetate; DMSO: Dimethyl sulfoxide; DSS: Dextran sodium sulfate; γ-GCSc: γ-glutamylcysteine synthetase catalytic subunit; γ-GCSm: γ-glutamylcysteine synthetase modifier subunit; GR: Glutathione reductase; HO-1: Heme oxygenase-1; Keap1: Kelch-like ECH-associated protein 1; MAPK: Mitogen-activated protein kinases; MTT: 3-(4,5-dimethylthiazol-2-yl)-2,5-diphenyltetrazolium bromide; NQO-1: NAD(P)H:quinone oxidoreductase; Nrf2: NF-E2-related factor 2; PI3K: Phosphatidylinositol 3-kinase; PKC: Protein kinase C; ROS: Reactive oxygen species; TR: Thioredoxin reductase; UGT1A1: UDP glucuronosyltransferase 1 family, polypeptide A1.

## Competing interests

The authors disclose no conflicts.

## Authors’ contributions

YY: Conception and design, acquisition of data, analysis and interpretation of data, writing of the manuscript. XC: Acquisition of data, analysis and interpretation of data. JY: Analysis and interpretation of data. RE: Statistical analysis. XS: Acquisition of data, analysis and interpretation of data, proof-reading of the manuscript. CH: Development of methodology, revision of the manuscript. ZY: Technical support, analysis and interpretation of data. XX: Conception and design, interpretation of data, revision of the manuscript. WL: Statistical analysis. PC: Obtained funding, conception and design, study supervision, revision of the manuscript. All authors read and approved the final manuscript.
